# Loading Patterns of Rubber-Based Resistance Bands across Distributors

**DOI:** 10.3390/sports7010021

**Published:** 2019-01-16

**Authors:** Alex D. Fuentes, Connor J. Smith, Todd C. Shoepe

**Affiliations:** Human Performance Laboratory, Department of Health and Human Sciences, Loyola Marymount University, Los Angeles, CA 90045, USA; alex.fuentes.us@gmail.com (A.D.F.); cee.smith12@gmail.com (C.J.S.)

**Keywords:** variable resistance, material properties, reliability, intra-repetition

## Abstract

Variable resistance implemented through concurrent use of rubber-based resistance bands and free weights is commonly used in training athletes. The purpose of this study was to examine the consistency of rubber-based resistance (RBR) band loading patterns across four distributors. Bands (*n* = 141) were obtained from online distributors (Rogue Fitness, EliteFTS, RubberBanditz, and Power Systems) across a spectrum of available widths (0.635, 1.270, 2.860, 4.450, 6.350, and 10.160 cm). At least five bands for each width were stretched in 5 cm increments from resting (100 cm) to twice resting length (200 cm) while tensile resistance was measured using a load cell integrated with a digital controller. Each band was tested twice on non-consecutive days producing an intertrial intraclass correlational coefficient (ICC) between 0.93–0.99 with a grand mean ICC across all repeated measures of 0.99. Statistical differences were observed in mean resistance for bands of equal thickness across distributors. Significant correlations were found between a range of tensile load expressed as a total load and band thickness (*r* = 0.658) and when expressed as a percentage (*r* = −0.386). This study is useful for strength and conditioning professionals and clinicians who should be cognizant of loading variability within both bandwidths and between distributors.

## 1. Introduction

The world of sports medicine frequently relies on the use of rubber-based resistance (RBR) bands in both performance and rehabilitation settings. Historically, competitive powerlifters are known to alter the kinetics of multi-joint exercises (e.g., squat, deadlift, bench press, and shoulder press) during training through the addition of RBR bands to traditional free-weight resistance modes [1,2,3]. Moreover, RBR bands have also been prominently used in clinical rehabilitation settings [4,5]. Combining RBR to free weight exercises produces a variable intra-repetition resistance [6] that alters the kinematics and kinetics of resistance exercise [7,8,9,10] in a way that is potentially more beneficial to strength and power than conventional loading paradigms [11,12]. The addition of RBR bands to a barbell exercise, for instance, theoretically provides progressively increasing resistance to match the ascending relationship between force generation and joint angle associated [13] with multi-joint lower extremity exercises [6].

Efforts have been made towards quantifying and characterizing variables related to stress-strain properties of RBR bands and loading properties of RBR materials for both therapeutic [14,15,16] and performance applications [17,18]. Separate works by Wallace et al. [9], Shoepe et al. [17], and McMaster et al. [18] have sought to predict a specific load at varying locations throughout an exercise’s range of motion in strength and conditioning settings. By examining changes in resistance when each band was stretched, these studies provided a methodology for quantifying RBR, which may be useful for prescribing specific loading intensities. McMaster et al. [18] described variability within a given band thickness that was deemed practically significant. However, this study only evaluated two bands per thickness and only within a single distributor or manufacturer. This documented yet incomplete understanding of loading variance could benefit in terms of proficiency or intensity and volume prescription as well as to the safety of client users. Differences between intended (prescribed) and actual loading due to inconsistencies in seemingly identical bands would negatively influence the relationship between training stimulus and adaptation. Safety could be reduced directly with bilateral exercises that simultaneously require two bands, which are potentially at opposite ends of the loading range. The two bands are inadvertently used. This would create an unintentional asymmetry with potentially damaging effects, particularly with near maximal efforts. 

Therefore, current literature provides minimal objective quantification for the consistency of RBR band resistance within and across distributors. The purpose of this study was to describe the consistency of a resistive force produced by RBR bands within and across distributors.

## 2. Methods

### 2.1. Experimental Approach

Because of the temperature and humidity of sensitive material properties of rubber elastic, all data collection for this study was conducted in the Applied Physiology Lab (APL), which is a temperature-controlled facility. A total of 141 RBR bands of varying widths and thicknesses with reported resting lengths of 100-cm from four different distributors (EliteFTS, London, OH, USA, Power Systems, Knoxville, TN, USA, RubberBanditz, Los Angeles, CA, USA, and Rouge Fitness, Columbus, OH, USA) were assessed. The full set of band thicknesses and distributors assessed for this investigation are provided in Table 1. The bands from Elite FTS were from their reported standard line and not their higher performance, vulcanized line of RBR bands. The two 1.270-cm band thicknesses represent both a smaller cross-sectional area (CSA) version (1.270 s) and a larger, thicker CSA version (1.270 t). All bands were ordered at the same time, received within a week of each other, and placed in the laboratory for at least six months prior to testing where they remained until data collection was completed. This was allowed for adequate temperature and humidity normalizations to occur equally for all bands, according to best practice guidelines for elastomer materials testing [19]. Unfortunately, due to the time intensive nature of the assessment protocol that took up 15 minutes per band, the analysis was completed over a six-month period of time. 

### 2.2. Procedures

**Figure 1:**
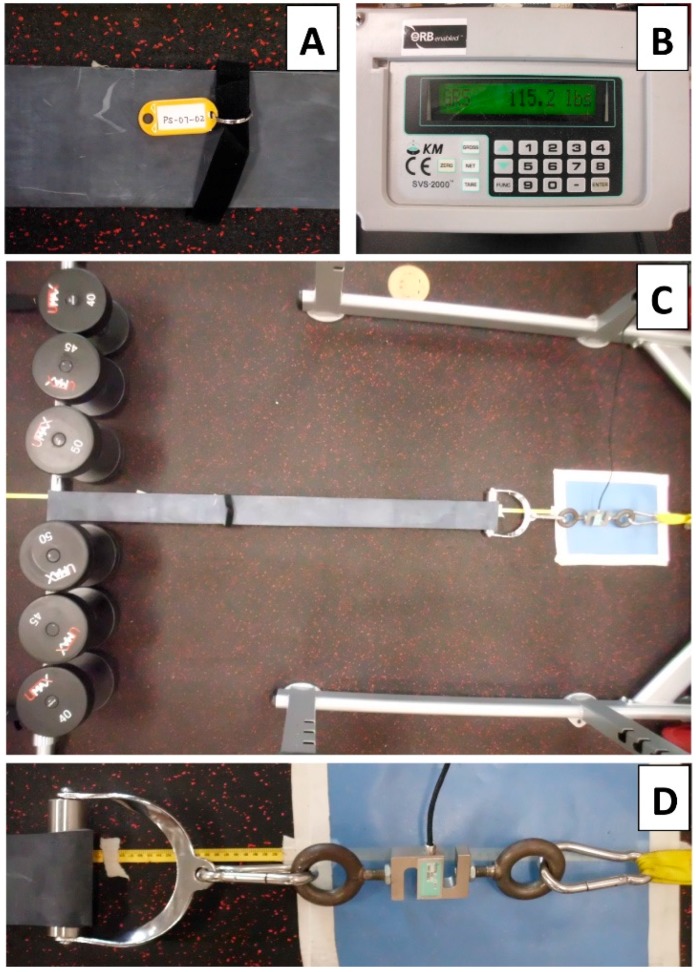
Experimental Set-up. (**A**) Band identification tags, (**B**) digital controller, (**C**) wide shot of band measuring apparatus, and (**D**) close-up of connections and load tensiometer.

The experimental configuration (Figure 1A–D) was modeled after the set up previously described by McMaster et al. [18]. The bands were each identified with a tag for consistent identification of distributor, thickness, and sample number (Figure 1A). The bands were wrapped around a standard Olympic barbell and anchored to a standard squat rack through a system of calipers and straps, including the load cell (Figure 1C). A smooth, custom-built handle was acquired that attached the band to the load cell in order to fit all band widths and thicknesses. A surface free of knurling was mandatory to allow the band to freely slide across the surface of the handle circumference as bilateral tensional forces (produced by the two sides of the band) reached self-equilibrium during the assessment protocol (Figure 1D). This also served to minimize mechanical damage to the bands over repeated trials. For the same reasons, the barbell itself, was likewise fitted with a smooth-surfaced, rotating, metallic sleeve over which the band was laid.

Tensile resistance was recorded via a load cell (DBBP-500, Kistler-Morse, Spartanburg, SC, USA) and displayed on a digital controller (SVS2000, Kistler-Morse, Spartanburg, SC, USA) in pounds, which were subsequently converted to kilograms (Figure 1B). Prior to each stretch, the load cell was unloaded and zeroed to ensure accuracy and consistency. To stretch the bands, the barbell was rolled to the desired distance and held in position by equally weighted dumbbells on either side (Figure 1D). For convenience, the total number and weight of the dumbbells depended on band resistance. Each band was systematically stretched to a distance twice its resting length of 100 cm in 5-cm increments to an overall length of 200 cm. This length was chosen due to previously published work identifying population-specific volume from concurrent free-weight and rubber-band resistance training, which revealed band length means of 86% of participant height during squat activities [20] and additional unpublished data from our lab showing band lengths well over twice the participant body height with a single-wrapped band attachment. This is described by Shoepe et al. [17]. Each increment of stretch for every band was measured from a tape measure affixed to the floor underneath the band being stretched. At each increment, the dumbbells were positioned and readings were taken after 3 to 5 seconds to allow for value stabilization. Each individual band was stretched twice over non-consecutive days to assess the intertribal reliability. Stiffness (stress/strain) was calculated in part with cross-sectional area measurements determined as the thickness and width of the band at rest. These were determined for every band experimentally through the mean of three successive height and width measurements with a 150-mm digital caliper (Sparkfun Electronics, Niwot, CO, USA) to the nearest 0.00001 inch.

### 2.3. Statistics

Intertrial reliability was assessed with two-way fixed, intraclass correlation coefficients (ICC) and the standard error of measurement (SEM) for each band thickness. One-way ANOVAs were performed to assess mean resistance production for each band width across the four distributors with Bonferoni’s procedure used for the post hoc analysis. Pearson product correlations were used to determine potential relationships between band thickness and tension ranges observed for all bands assessed (both mass and as a percentage). A further one-way ANOVA was used to examine the potential differences in tension ranges observed as both mass and as a percentage, for the five thicknesses that were assessed equally from all distributors (e.g., 0.635, 1.270 s, 1.270 t, 2.860, 4.450, 6.350, and 10.160 cm). Statistical significance for all tests was set at *p* < 0.05. All ICCs and SEMs were completed with a customized spreadsheet application (Excel for Mac 2011, version 14.6.3, IBM, Redmond, WA, USA) with ANOVAs and correlational analysis completed with SPSS for Mac (IBM SPSS Statistics 2013, version 22.0.0.0, IBM Corp, Armonk, NY, USA). 

## 3. Results

A high degree of reliability was found between repeated measurements across all band thicknesses (Table 2). No thickness by distributor ICC was less than 0.93 (observed in Distributor C for the 0.635 thickness) with a 95% confidence interval from 0.905 to 0.952 (*F* (125,125) = 28.5, *p* < 0.0001). The grand mean of all measurements across bands and distributors produced an ICC of 0.99 with a 95% confidence interval from 0.99 to 0.99 (*F* (2981,2981) = 1676.9, *p* < 0.0001).

**Figure 2:**
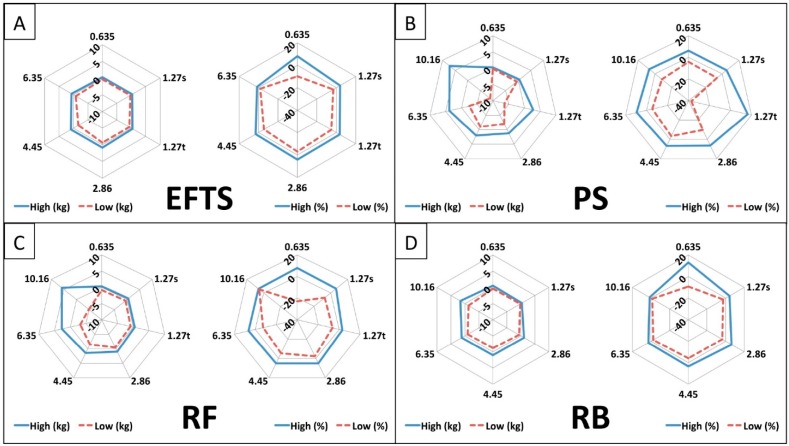
(**A**–**D**) Range plots of resistance by band and distributor. For each figure, the zero value represents the mean for each subset of bands assessed. Range of values observed is displayed between the dashed and solid lines, which represent the minimum and maximum values observed for each subset of bands, respectively. Band loading ranges for every distributor are expressed, on the left, in absolute (kg) and, on the right, in relative (%) units at a length of 200 cm. The x-axis values are reported thicknesses provided in centimeters. EFTS = EliteFTS, PS = Power Systems, RB = RubberBanditz, RF = Rogue Fitness.

Significant differences between tensile resistance and distributor were seen at all but one thickness (Table 2). There was a significant effect of the independent variable known as the distributor on the dependent variable of tensile resistance at 200 cm for band thicknesses of 0.635 cm at the *p* < 0.05 level [*F* (3,20) = 6.35, *p* = 0.004], 1.270 s cm at the *p* < 0.05 level [*F* (3,19) = 31.80, *p* < 0.001], 1.270 t cm at the *p* < 0.05 level [*F* (2,19) = 11.66, *p* = 0.01], 2.860 cm at the *p* < 0.05 level [*F* (3,22) = 14.26, *p* < 0.001], 6.350 cm at the *p* < 0.05 level [*F* (3,22) = 5.34, *p* = 0.008], 10.160 cm at the *p* < 0.05 level [*F* (2,17) = 8.43, *p* = 0.004]. No significant differences were seen of the independent variable called the distributor on the dependent variable of tensile resistance at 200 cm for band thickness of 4.450 cm at the *p* < 0.05 level [*F* (3,22) = 2.87, *p* = 0.65]. Absolute (kg) and relative (%) ranges of tension at 200 cm are provided in Figure 2. Each distributor is depicted individually by the mean high (solid lines) and low (dashed lines) of each group of band thicknesses.

There was a significant positive correlation between band thickness and range of tensile resistance (expressed as kg) measured at 200 cm, *r* = 0.658, *n* = 28, and *p* < 0.0001 while a significant negative correlation was seen between band thickness and the range of tensile resistance when expressed as a percentage of resistance measured at 200 cm, *r* = −0.386, *n* = 28, *p* = 0.021. 

There was a significant effect of the independent variable referred to herein as *thickness*, on the dependent variable referred to herein as a *range of tensile resistance* at 200 cm at the *p* < 0.05 level [*F* (4,20) = 4.88, *p* = 0.01]. A post hoc analysis using the Bonferoni method revealed that the range of tensile resistance for the 0.635 cm thickness (*µ* = 0.81, SD = 0.29) and the 1.270 s (*µ* = 0.84, SD = 0.27) were both significantly less than 4.450 cm (*µ* = 2.70, SD = 0.37) and 6.350 cm (*µ* = 3.97, SD = 2.51). Additionally, the 2.860 cm range (*µ* = 1.98, SD = 0.85) was less than the range of the 4.450 cm band.

## 4. Discussion

This study demonstrates the existence of measurable and significant inconsistencies in the RBR band mean resistance between RBR band distributors when statistical differences were observed in the mean resistance for bands of equal thickness across distributors. However, the purpose of this study was not to call attention to inconsistencies within any one or more distributors, but instead to provide insight for clinicians and strength and conditioning professionals regarding observable and practical differences in RBR band resistance. This data did not demonstrate clear identification of systematically elevated or lowered band resistances of a given thickness between manufacturers. Furthermore, attributing responsibility to distributors potentiates negligence as an important note, which is that the nature of this investigation does not permit the identification of whether manufacturing, storage, handling, or aging are responsible for the variability identified within band thickness nor between distributors. However, four individual bands in particular, contributed to larger ranges within a given thickness. Of these, three (1.270 t, 6.350, 10.16) were observed in bands acquired from PS with the other obtained from RF (6.350). The occurrence of notable “outlier” bands may be most relevant to practitioners where a greater mismatch of expected and actual load may complicate exercise programming. Given the relatively small sample size of each thickness within a distributor, generalizations regarding consistency attributed to the distributor should be cautioned. Instead, it is more important that practitioners are aware that outliers exist in commercially available RBR bands so that they might anticipate their occurrence, mark them for future use, and adjust programming variables accordingly. 

Previously, McMaster et al. [18] demonstrated non-statistical but practical differences between a pair of bands from an online distributor not investigated in this study. They reported a mean difference of 4.9 kg of resistance at an elongation of twice resting length for bands of 0.48 m thickness. Differences among other band thicknesses were considerably lower, which speaks to the possibility that this large variability was due to the occurrence of a potential outlier. One major purpose of the present study was to expand on this work by increasing the sample size of each thickness in order to account for occasional outliers on group thickness means. The only thicknesses that produced resistance differences on the order seen by McMaster et al. [18] were bands distributed from EFTS at the 1.270 t and 10.160 thickness where large variability and ranges were observed (1.270 cm, range = 10.7–19.8 kg, 10.160 cm, kg, 97.8–113.9 kg). Additionally, bands distributed from Rubberbanditz demonstrated large variability at the thickest band (10.160 cm, range = 91.0–101.7 kg). 

Single bands that deviated significantly from the group mean largely influenced the occasional thicknesses that displayed a very large variability. However, ranges at all thicknesses were around 2% or higher, which is a value that could have a profound effect on the expected resistance an exerciser might encounter and what would actually be provided.

The present finding of an extreme range of −37% to 16% for bands of similar thickness is not unreasonable when compared to previous work showing ranges of −3% to 35% in commercially available RBR bands [16]. However, while the bands of Thomas et al. were made of similar material, their structural composition is different in that they were hollow and cylindrical, which both constitute morphological properties that could affect tensile characteristics.

Nonetheless, the wide ranges seen in commercially available RBR bands are noteworthy from a practical sense. As an example to illustrate this point, using 6.350 cm bands would produce a range of actual resistances at twice resting length equal to up to 7 kg for each of the two bands (14 kg total) for the barbell squat exercise. Further concern is produced if one of the bands were to express loading at the lower range and the second band were to exhibit loading behavior near the upper end of the range, which creates disproportionate, imbalanced loading that could affect desired adaptations and a potential for injury.

**Figure 3:**
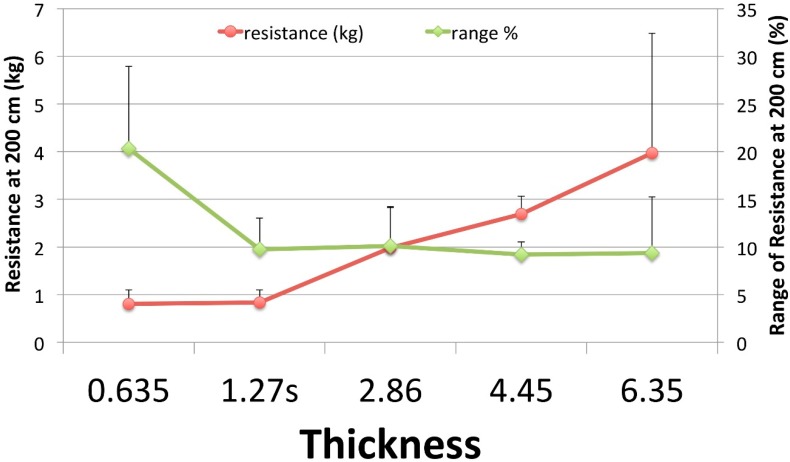
Range of Resistances. Shown are the ranges of resistances observed at twice resting length (200 cm). These are expressed in both absolute (kg) and relative (%) resistance. EFTS = EliteFTS, PS = Power Systems, RB = RubberBanditz, RF = Rogue Fitness.

Mean resistance at twice the resting length of the bands varied significantly across distributors with the exception of bands of the 4.450-cm thickness. It is noteworthy that an inverse relationship was found between the ranges of absolute and relative resistance. As band thickness increased, the range of resistance in kilograms increased and the range of resistance in percentage from the mean decreased (Figure 3). The resistance range increases with increasing thickness, which has been previously reported with thinner rehabilitative RBR bands (Thera-bands™, Akron, OH, USA) [14,15]. This is likely because the resistance-producing capability of thinner bands is less than that of thicker bands, which allows for a smaller range of mean band resistances. The implications in practice are that, at the lowest band thicknesses with expected low absolute loading (e.g. 1 kg), the actual loading would be expected to vary only by 0.2 kg, but this represents a quantity of as much as 20%. The reverse is true for the highest band thickness with expected high absolute loading (e.g., >50 kg), where the actual loading would be expected to vary up to 4 kg, but this would represent only 10% of the expected value.

Future research should focus on the changing loading properties of RBR bands over time. Elastomers such as RBR show sensitivity to repeated stress and rate of loading [19]. In contrast to the present study, while investigating thinner rehabilitative bands (Thera-bands, Akron, OH, USA), which produce less tensile resistance, Simoneau et al. [14] have previously shown that RBR bands demonstrate alterations in tensile loading characteristics following 500 loading cycles. These differences were more pronounced when assessing shorter elongations and in thicker bands. The authors suggested that most of the material fatigue occurs in the first 50 cycles and fatigue occurs more rapidly with greater elongations. This is noteworthy since significant and rapid fatigue of RBR bands in shorter excursions would affect exercise applications to a great extent. Shorter elongations occurring in the elastic region of the stress-strain relationship of these bands are likely common in resistance training applications. Further investigation into whether the material fatigue findings of Reference [14] would be replicated for thicker bands used in performance settings, such as in the present study, is warranted. 

Additionally, although all of these bands are seemingly similar in all ways with the exception of color and thickness, it is unknown whether differences exist in the composition, manufacturing practices, and or subtle morphometry. Future studies are warranted to examine the chemical composition, effects of the manufacturing practices, and micro-architecture of the bands.

The principle limitation of the study was the assessment timeframe, which took approximately six months (June to December). This indicates the potential time-related effects on rubber material. As previously mentioned, efforts were made to reduce environmental variability by storing the bands in the APL, which contains a thermostat-regulated unit set to between 68–72° throughout the duration of the study. Bands were assessed within the first distributor and second within thickness while efforts were made to complete the second, reliability trial in close proximity to the original assessment. While stretching the thicker, stronger bands, the apparatus used to affix the load cell to the squat rack was checked between incremental measurements for slippage during the greater and more forceful elongations. The load cell was always aligned with the 100-cm mark on the tape measure at the start of each individual band assessment, and, as needed, would be adjusted over time to promote accurate resting length assignments. In addition, alignment with the tape measurer for each stretch was visually estimated, which possibly affects the resistance measurement especially at greater lengths (i.e., the stronger the band, the more sensitive resistance became with each centimeter). However, following the recommendations of McMaster et al. [18] to use more frequent, shorter increments, this effect was minimized by using 5-cm increments, which allow for the collection of 11 measurements per band trial from 100 to 200 cm.

## 5. Conclusions

As a result of this study, inconsistencies in RBR band mean resistance have been identified and quantified across four major RBR band distributors. For practical application purposes, resistance training professionals and clinicians should be aware that RBR bands of similar reported dimensions might vary in mean resistance. Because the bands used in this analysis were obtained anonymously via online distributors, it is not possible to discern whether the inconsistencies found were the result of manufacturing or handling procedures. Examination of individual RBR band material properties as well as production, transportation, and storage practices might explain mean resistance inconsistencies within and across distributors. Nonetheless, it is likely that a collection of bands seemingly identical in distributor, color, and thickness will produce predictable and notable inconsistencies in loading patterns when used in applied settings. This could affect not only the appropriate training stimulus, but safety if bilateral banding is required for a particular exercise. One recommendation for practitioners to consider given this conclusion is to uniquely label each band in the facility so that consistency of usage can be employed in order to mitigate potential discrepancy in volume and intensity for athletes and patients. 

## Figures and Tables

**Table 1:** The Distributors and Band Thicknesses Assessed. EFTS = EliteFTS, PS = Power Systems, RB = RubberBanditz, RF = Rogue Fitness.

Band Thickness (cm)	EFTS	PS	RB	RF
0.635	Y	Y	Y	Y
1.270 s	Y	Y	Y	Y
1.270 t	Y	Y		Y
2.860	Y	Y	Y	Y
4.450	Y	Y	Y	Y
6.350	Y	Y	Y	Y
10.160		Y	Y	Y

**Table 2:** Band Property Variables Across Thickness and Distributor. EFTS = EliteFTS, PS = Power Systems, RB = RubberBanditz, RF = Rogue Fitness, SD = standard deviation, CSA = cross-sectional area. * represents statistical difference from distributor EFTS. ^†^ represents the statistical difference from distributor PS. ~ represents the statistical difference from distributor RB. Statistical difference set at *p* < 0.05.

Thickness (cm)	Distributor (*n*)	Resistance (mean ± SD, kg)	Range (kg)	ICC	SEM (kg)	CSA (cm^2^)	Stiffness (MPa)
**0.635**	**EFTS** (5)	3.8 ± 0.3	3.4–4.1	0.94	0.3	0.29	4.3
	**PS** (5)	4.6 ± 0.2 *	4.4–4.9	0.99	0.1	0.28	6.6
	**RB** (6)	4.1 ± 0.3	3.7–4.6	0.93	0.3	0.29	5.7
	**RF** (5)	3.9 ± 0.5 †	3.0–4.2	0.99	0.5	0.30	5.3
**1.27 s**	**EFTS** (5)	9.6 ± 0.3	9.3–10.0	0.99	0.3	0.64	5.5
	**PS** (5)	8.6 ± 0.4 *	8.1–9.1	0.98	0.4	0.57	5.4
	**RB** (6)	8.0 ± 0.2 *†	7.8–8.3	0.98	0.3	0.57	5.6
	**RF** (6)	8.3 ± 0.4 *†	7.7–8.8	0.99	0.2	0.59	5.8
**1.27 t**	**EFTS** (5)	13.1 ± 0.5	12.4–13.6	0.99	0.2	0.90	3.9
	**PS** (5)	17.0 ± 3.9 *	10.7–19.8	0.99	0.2	0.82	6.9
	**RB** (0)						
	**RF** (6)	11.6 ± 0.6 †	11.1–12.4	0.99	0.1	0.88	5.1
**2.86**	**EFTS** (5)	20.9 ± 0.6	20.3–21.8	0.99	0.3	1.37	3.4
	**PS** (5)	20.2 ± 1.2	18.2–21.4	0.99	0.2	1.34	4.8
	**RB** (6)	18.0 ± 0.7 *†	17.3–19.1	0.99	0.3	1.36	5.1
	**RF** (6)	19.4 ± 0.6 *~	19.0–20.4	0.99	0.3	1.30	5.8
**4.45**	**EFTS** (5)	30.0 ± 1.0	28.3–30.9	0.99	0.4	2.06	3.7
	**PS** (5)	29.9 ± 1.2	28.9–31.9	0.99	1.5	2.07	4.7
	**RB** (6)	29.2 ± 1.0	28.0–30.2	0.99	0.2	2.04	5.4
	**RF** (6)	28.3 ± 1.1	26.8–29.8	0.99	0.4	2.04	4.1
**6.35**	**EFTS** (5)	44.6 ± 0.6	43.8–45.3	0.99	0.5	2.97	3.5
	**PS** (5)	41.2 ± 2.4 *	38.7–45.1	0.99	0.3	2.75	5.4
	**RB** (6)	40.9 ± 0.9 *	39.9–42.1	0.99	0.3	2.96	4.7
	**RF** (6)	42.3 ± 2.0	39.1–45.0	0.99	0.4	2.86	2.9
**10.16**	**EFTS** (0)					
	**PS** (5)	106.7 ± 6.7	97.8–113.9	0.99	2.3	6.49	5.1
	**RB** (6)	99.5 ± 1.4 †	98.2–101.1	0.99	0.9	6.72	5.7
	**RF** (6)	95.9 ± 4.0 †	91.0–101.7	0.99	1.5	6.54	5.2
